# Benefits of Regular Intake of Glucolacto-Oligosaccharides on Gut Health in Adults with Low Defecation Frequency: A Randomized, Double-Blind, Placebo-Controlled Study

**DOI:** 10.3390/microorganisms14050955

**Published:** 2026-04-23

**Authors:** Yuichi Yoshizawa, Junya Ishida, Atsushi Shimonaka, Satoshi Hanamura, Akika Nagira, Mami Minakata, Akiko Koizumi, Aoi Fujieda, Hiroki Negishi, Shigenori Kanemura, Seiya Makino, Kenichi Hojo

**Affiliations:** 1 Health Science Research Unit, Division of Research and Development, Meiji Co., Ltd., Tokyo 192-0919, Japan; yuuichi.yoshizawa@meiji.com (Y.Y.); atsushi.shimonaka@meiji.com (A.S.); akika.nagira.aa@meiji.com (A.N.); aoi.fujieda@meiji.com (A.F.); seiya.makino@meiji.com (S.M.); kenichi.houjou@meiji.com (K.H.); 2 Lactic Acid Bacteria & Fermentation Technology Research Unit, Division of Research and Development, Meiji Co., Ltd., Tokyo 192-0919, Japan; satoshi.hanamura@meiji.com (S.H.); mami.minakata@meiji.com (M.M.); akiko.koizumi@meiji.com (A.K.); 3 Wellness Science Laboratory, Meiji Co., Ltd., Tokyo 192-0919, Japan; hiroki.negishi.aa@meiji.com; 4 Analytical Science Research Unit, Division of Research and Development, Meiji Co., Ltd., Tokyo 192-0919, Japan; shigenori.kanemura@meiji.com

**Keywords:** glucolacto-oligosaccharides, galactosylkojibiose, prebiotics, *Parabacteroides*, alpha diversity, gut health, randomized controlled trial

## Abstract

The significance of less abundant genera within the gut microbiota, such as *Parabacteroides*, remains largely unexplored. Despite its low levels, *Parabacteroides* is highly conserved and potentially beneficial across populations. This trial aimed to evaluate whether a four-week intake of glucolacto-oligosaccharides (GLO), previously reported as an enhancer of *Parabacteroides*, improves defecation frequency as the primary outcome. It also assessed holistic gut health and underlying microbiota-based mechanisms. In this randomized, double-blind, placebo-controlled trial, 50 healthy Japanese participants with a defecation frequency of five or fewer times per week were enrolled. The mean (±SE) weekly defecation frequency in the GLO group was 3.2 ± 0.2 at baseline, increasing to 5.8 ± 0.6 at week 4, whereas that in the placebo group was 3.4 ± 0.3 at baseline, increasing to 4.4 ± 0.3 at week 4. The time-dependent weekly defecation frequency was significantly higher in the GLO group than in the placebo group (*p* = 0.029). Changes in the relative abundance of the genus *Parabacteroides* significantly increased in the GLO group compared with in the placebo group. Changes in fecal bile acid composition were also confirmed in the GLO group compared with the placebo group, which was thought to be due to the unique features of *Parabacteroides*. Furthermore, changes in alpha diversity indices were significantly higher in the GLO group than in the placebo group (Simpson, *p* = 0.041; Pielou, *p* = 0.022). Additional analysis demonstrated that the increase in alpha diversity in the GLO group was significantly correlated with the increase in the relative abundance of *Parabacteroides* (*p* = 0.006), which tended to be associated with decreases in serum gamma-glutamyltransferase (*p* = 0.089) and serum triglyceride (*p* = 0.075) levels. These data suggest that GLO intake improved defecation status, selectively increased *Parabacteroides*, and harmonized the gut environment.

## 1. Introduction

The human gut microbiota comprises more than 130 genera and 3000 species, and its composition is partly characterized by ethnicity and dietary habits [1,2,3]. For example, *Bifidobacterium* and *Prevotella* are commonly found in higher proportions in Asians, whereas *Bacteroides* and *Ruminococcus* tend to predominate in Westerners [4,5,6]. Thus far, the roles of certain types of predominant bacteria, such as *Bifidobacterium*, have been well established; however, recent studies have also highlighted the unique roles of less abundant or rare bacteria [7]. *Parabacteroides* is one such rare microbiota, with lower abundance than predominant bacteria such as *Bifidobacterium*, but it commonly comprises a small percentage of the gut microbiota in diverse populations [8]. Thus, *Parabacteroides* may play a conserved role in human health. In fact, a decreased abundance of *Parabacteroides* has been reported in individuals with multiple sclerosis [4], non-alcoholic fatty liver disease (NAFLD) [5], obesity [6], and metabolic syndrome [7] compared with healthy individuals. Moreover, *Parabacteroides* has been reported to be linked to the maintenance of intestinal barrier integrity in older adults, thereby supporting healthy aging [9]. In centenarians, *Parabacteroides* has attracted attention as a producer of rare bile acids that are associated with longevity [10]. *Parabacteroides* is abundant in centenarians and decreases with age in ordinary people [10,11], highlighting the importance of strategies to maintain or enhance its levels.

Prebiotics are defined as “non-digestible compounds that beneficially affect the host by selectively stimulating the growth and/or activity of specific bacteria in the colon, thereby improving host health.” [12,13,14]. Established prebiotics include oligosaccharides, dietary fibers, and resistant proteins. The health benefits of prebiotics include improved bowel movement [15], regulation of immune functions [16], protection of the intestinal barrier [16,17], anti-inflammatory actions [18], and improvement in glucose and lipid metabolism [19,20]. Classically, prebiotic effects are mediated by increases in *Bifidobacteria* and *Lactobacilli* (producers of lactic acid) or by the stimulation of butyrate-producing bacteria. On the other hand, our previous in vitro study demonstrated that a component of glucolacto-oligosaccharides (GLO) has a unique capacity to preferentially increase the growth of *Parabacteroides* [21]. GLO is material that has been successfully industrialized through our unique manufacturing method. Their primary ingredient is permeate, a byproduct generated during the manufacturing process of cheese, cream, and milk or whey protein from raw milk. While GLO itself has resource value, their functional roles in human health remain unclear.

Constipation is one of the most common digestive problems and is characterized by difficult, unsatisfactory, or infrequent defecation. It affects approximately 10–20% of the global population [22]. In addition, individuals with chronic constipation report a lower quality of life (QOL) and work productivity than those without the condition [23]. Thus, bowel dysfunction affects not only physical but also mental and social well-being. This randomized, double-blind, placebo-controlled, parallel-group clinical study was designed to initially assess the effects of GLO on gut health as a basic prebiotic function, prior to evaluating its more advanced health benefits. Since *Parabacteroides* is known to increase certain bile acids [10], we hypothesized that *Parabacteroides*-induced changes in bile acid composition could serve as a chemical stimulus, thereby improving bowel function. We set improvement in defecation status, a representative indicator of gut health, as the primary outcome, and we also investigated the potential mechanisms of action through modulation of the gut microbiota as a secondary outcome in a four-week intervention trial.

## 2. Materials and Methods

### 2.1. General Characteristics of GLO

GLO contains substrates that we have long sought to promote the growth or activation of rare beneficial microbiota, rather than abundantly studied microbiota, in order to understand the significance of the microbial diversity of our gut. Through in vitro fecal culture and monoculture experiments, we identified a unique substrate that promoted a relative increase in *Parabacteroides*. GLO is a general term for oligosaccharides comprising trisaccharides (4-galactosylkojibiose, 4-GK) and tetrasaccharides (glucosyl-galactosylkojibiose, GGK) (see Supplemental Figure S1 for details). Our data showed that 4-GK and GGK had comparable capacities to increase *Parabacteroides* (see Supplemental Figure S2 for details). GLO syrup was enzymatically synthesized from lactose, which is the main component of the byproduct permeate, and sucrose using dextransucrase from *Liquorilactobacillus satsumensis*. In this study, the GLO produced from refined lactose and sucrose was purified by chromatography. The amount of GLO was quantified as 1.4 g per pack using quantitative NMR.

### 2.2. Study Design

A randomized, double-blind, placebo-controlled, parallel-group trial was conducted by assigning participants to either a GLO or placebo group. This study was approved by the Institutional Review Board (approval number: 24081601) of Chiyoda Paramedical Care Clinic (Tokyo, Japan) and was conducted from September to November 2024 at Chiyoda Paramedical Care Clinic (Tokyo, Japan). In compliance with the ethical principles of the Declaration of Helsinki and the ethical guidelines for epidemiological studies, participants were fully informed of the purpose and content of the study, and written informed consent was obtained from all participants before participation. The study protocol was registered on 30 August 2024, using the University Hospital Medical Information Network (UMIN) Clinical Trial Registration System (UMIN000055373). The fecal samples collected in the study (UMIN000055373) were secondarily used to examine the relative abundance of bile acids. This additional study was approved by the Institutional Review Board (approval number: 247) of Meiji Institutional Review Board (Tokyo, Japan), registered on 1 August 2025 (UMIN000058596), and was conducted at Meiji Co., Ltd. (Tokyo, Japan).

### 2.3. Participants

Healthy individuals who met all of the following inclusion criteria and none of the following exclusion criteria were enrolled. The inclusion criteria were: (1) healthy Japanese males and females aged 20 to 64 years at the time of informed consent; (2) an average defecation frequency of five or fewer times per week during the two months before consent; and (3) the ability to fully understand the purpose and procedures of the study, provide voluntary informed consent, and sign the consent form. The exclusion criteria were: (1) regular consumption (three or more times per week) of Foods for Specified Health Uses (FOSHU), Foods with Functional Claims, or health supplements (including products affecting intestinal regulation, probiotics, or prebiotics) that could interfere with the study; (2) inability to discontinue intake of these foods or supplements from the time of consent onward; (3) use of medications that could affect the study (e.g., antibiotics, intestinal regulators, or laxatives) within one month before the intervention or planned use during the study period; (4) inability to abstain from alcohol from the day before the preliminary examination or each test; (5) excessive alcohol intake; (6) smoking or cessation of smoking within the last six months; (7) gastrointestinal diseases or history of gastrointestinal surgery (excluding hemorrhoidectomy) that could affect digestion or absorption; (8) current treatment for lifestyle-related diseases or other medical conditions; (9) ongoing medical interventions, such as dialysis, exercise therapy, or dietary therapy; (10) irregular sleep–wake cycles (e.g., shift workers or night shift workers); (11) planned travel or relocation for three nights and four days or longer during the study period; (12) participation in other clinical trials, prior participation within four weeks before the current trial, or planned participation during the study period; (13) pregnancy, lactation, or planned pregnancy during the study period; (14) self-reported irritable bowel syndrome (IBS); (15) self-reported history of excessive abdominal symptoms (e.g., severe diarrhea, and bloating) upon oligosaccharide intake; (16) food allergies; (17) blood donation exceeding the specified volume within the defined period; and (18) any other condition deemed inappropriate for study participation by the principal investigator. Additionally, strict dietary restrictions were not imposed in this study.

A third-party allocation manager randomly assigned the participants to two groups—one receiving the test food and the other receiving the placebo food—using a computer-generated randomization sequence. Given the lack of pilot studies on the effects of GLO on defecation frequency, the sample size was determined with reference to previous studies that evaluated defecation frequency using similar oligosaccharide interventions [24,25,26]. Based on a previous study employing a parallel-group design [24], the required sample size was calculated with an alpha level of 5%, a beta of 20%, and an assumed effect size (Cohen’s *d*) in the range of 0.98–2.08. As a result, the necessary sample size was estimated to be 4.6 to 17.2 participants. In addition, for the human study investigating the effects of GLO [21], 25 participants per group were chosen to enable the assessment of its impact on the microbiota. Age, sex, and defecation frequency were used as allocation factors. The placebo group was instructed to ingest 30 g of water containing fructose and glucose, and the GLO group was instructed to ingest 30 g of water containing 1.4 g of GLO per serving. The ingredients per serving of placebo and GLO are presented in Supplemental Table S1. After the allocation manager confirmed that they were indistinguishable in flavor and appearance, the participants ingested one of the assigned test foods per day for 4 weeks, with no limit on the time of intake. The allocation manager, group assigner, and personnel responsible for test food coding worked independently. Thus, the participants and study staff were blinded to the test foods unless the three pieces of information necessary for revealing identity were collected. Blinding was maintained until fixation.

### 2.4. Defecation Status Questionnaires

The participants were instructed to record their daily defecation status, including defecation frequency, number of defecation days, stool volume, and stool consistency, for 1 week before test food intake and 4 weeks during the intake period. The participants were also instructed to record consumption of the test foods, changes in daily life regarding meal frequency, alcohol consumption, exercise, sleep duration, physical condition, and medication.

### 2.5. Fecal Sampling and Analysis

All fecal samples collected before intake and at the end of intake were subjected to NGS-based 16S rRNA bacterial profiling. Bacterial DNA was extracted from the feces, and amplicon sequencing analysis was performed at TechnoSuruga Laboratory Co., Ltd. (Shizuoka, Japan). The V3–V4 region of the 16S rRNA was amplified using region-specific primers (Pro341F–Pro805R) [27]. Sequence data were analyzed using Quantitative Insights into Microbial Ecology (QIIME2) version 2023.2. software [28]. Raw reads were quality-filtered and denoised using DADA2 version 4.3.1 [29]. Based on the minimum number of denoised reads among the samples, all samples were rarefied to 12,500 reads for standardization. Taxonomic assignment was performed using the SILVA 138 database [30]. Alpha diversity indices were used to assess the diversity of the gut microbiota in groups that ingested either the test food or the placebo food. Functional gene abundances were inferred from 16S rRNA gene sequence data using PICRUSt2 (v2.4.1) [31]. The predicted functional abundances were normalized to relative abundance. The concentration of each bile acid was calculated as its relative abundance of the total measured bile acids.

### 2.6. Quantitative Real-Time PCR Analysis of Parabacteroides Distasonis and Merdae

To assess the relative abundance of *P. distasonis* and *P. merdae*, species-specific primers targeting the 16S rRNA gene were employed. A list of the primer sequences used in this study is provided in Supplemental Table S2. Each reaction had a total volume of 20 µL, consisting of 10 µL of PowerUP^TM^ SYBR^®^ Green Master Mix (Thermo Fisher Scientific, Waltham, MA, USA), 0.1 µL of each primer, 4 µL of template DNA, and 5.8 µL of nuclease-free water. The qPCR thermal profile included an initial denaturation step at 95 °C for 2 min, followed by 40 amplification cycles comprising denaturation at 95 °C for 15 s and annealing/extension at 60 °C for 1 min. All reactions were run in duplicate. Negative controls (no template DNA) and a standard curve using serial dilutions of genomic DNA of each species were included. The genomic DNA of *Bifidobacterium longum* was used to generate a standard curve for total bacteria. The relative abundance of each species was calculated as the number of cells of the species divided by the total bacteria.

### 2.7. Blood Test

Blood samples were collected before and after the end of the intake period. The blood tests conducted by BML, Inc. (Tokyo, Japan) were as follows: hematologic test (White Blood Cell [WBC], Red Blood Cell [RBC], Hemoglobin [Hb], Hematocrit [Ht], Platelet [Plt]) and biochemical test (Total Protein [TP], Albumin [Alb], Total Bilirubin [T-Bil], Alkaline Phosphatase/International Federation of Clinical Chemistry and Laboratory Medicine [ALP/IFCC], Lactate Dehydrogenase/International Federation of Clinical Chemistry and Laboratory Medicine [LD/IFCC], Aspartate aminotransferase [AST], Alanine aminotransferase [ALT], Gamma-glutamyltransferase [γ-GT], Creatine Kinase [CK], Total Cholesterol [T-Cho], Triglyceride [TG], High Density Lipoprotein Cholesterol [HDL-C], Low Density Lipoprotein Cholesterol [LDL-C], Urea Nitrogen [UN], Creatinine [CRE], Uric Acid [UA], Sodium [Na], Potassium [K], Chlorine [Cl], Calcium [Ca], Glucose [GLU], Hemoglobin A1c (National Glycohemoglobin Standardization Program) [HbA1c (NGSP)]).

### 2.8. Measurements of Fecal Bile Acid

Bile acids were extracted from the fecal samples using a two-step methanol extraction method [32]. Briefly, 100 mg of fecal sample was homogenized with 0.85 mL of 50 mM sodium acetate buffer (pH 7.0) and an internal standard (CA-d4; Cayman Chemical Company, Ann Arbor, MI, USA) using a FastPrep system (6 m/s, 40 s, 2 cycles; MP Biomedicals, Santa Ana, CA, USA). The homogenate was further extracted with methanol, followed by sonication and heat treatment (85 °C, 30 min). After centrifugation, the combined supernatants were dried under nitrogen and reconstituted in 250 µL of 50% acetonitrile. An analysis was performed using LC-MS/MS (Xevo TQ-S micro; Waters, Milford, MA, USA) with an ACQUITY UPLC BEH C18 column (2.1 mm × 100 mm; Waters). The mobile phase consisted of (A) 0.01% formic acid and (B) acetonitrile:methanol (85:15 *v*/*v*). The column temperature was maintained at 45 °C with a flow rate of 0.45 mL/min. The gradient program was as follows: 0.5 min, 95% A; 1 min, 65% A; 4.5 min, 70% A; 6 min, 60% A; 8 min, 55% A; 11.5 min, 30% A; 12.15 min, 0% A; 15.5 min, 95% A; 18 min, 95% A. The injection volume was 5 μL. The following bile acids were analyzed: cholic acid (FUJIFILM Wako Pure Chemical Corporation, Osaka, Japan), deoxycholic acid, lithocholic acid, taurocholic acid sodium hydrate, glycocholic acid, ursodeoxycholic acid, chenodeoxycholic acid, sodium taurodeoxycholate hydrate, sodium taurochenodeoxycholate hydrate, and sodium glycodeoxycholate (all from Sigma-Aldrich, St. Louis, MO, USA); taurolithocholic acid sodium salt, ursocholic acid, and isodeoxycholic acid (all from Toronto Research Chemicals, North York, ON, Canada); glycolithocholic acid sodium salt, isolithocholic acid, 3-oxolithocholic acid, isoallolithocholic acid, and CA-d4 (all from Cayman Chemical Company); glycoursodeoxycholic acid (Tokyo Chemical Industry, Tokyo, Japan); and tauroursodeoxycholic acid sodium salt (Matrix Scientific, Columbia, SC, USA).

### 2.9. Statistical Analysis

Between-period comparisons with the baseline were performed using a Wilcoxon signed-rank test for bacterial abundance, bile acid, and the relative abundance of predicted 5α-reductase and using a paired *t*-test for alpha diversity. Between-group comparisons were performed as follows: Defecation status was evaluated using a mixed-effects model for repeated measures (MMRM) to assess changes over five time points. Changes in bacterial abundance and bile acids and changes in the relative abundance of predicted 5α-reductase were examined using the Mann–Whitney U test. Changes in alpha diversity were examined using *t*-tests after normality was confirmed. Regarding baseline characteristics, Fisher’s exact test was used for gender, the Mann–Whitney U test for age, and unpaired *t*-tests for BMI and defecation status. Unless otherwise specified, data are expressed as means ± standard error, and all statistical analyses were conducted using two-tailed tests. The significance level was set at 5%. The correlation between the relative abundance of *Parabacteroides* and other parameters was analyzed using the Pearson and Spearman correlation coefficient tests. Data analysis was conducted using R version 4.4.1, RStudio version 2024.04.2 (RStudio, Posit Software, Boston, MA, USA), and SPSS version 30.2 (IBM, Armonk, NY, USA).

## 3. Results

### 3.1. Participant Flow and Baseline Characteristics

**Figure 1:**
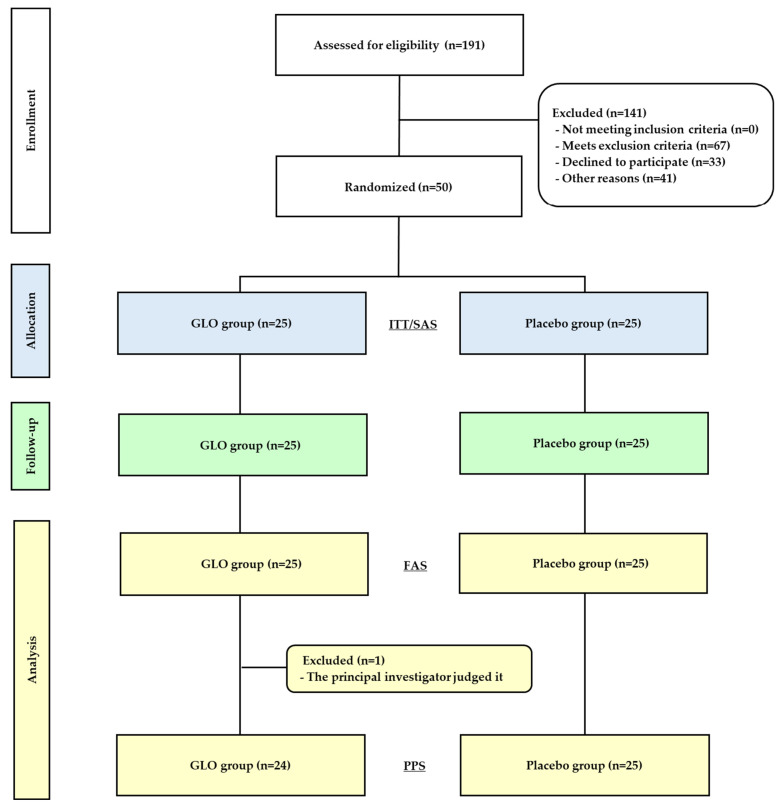
Flowchart of study participants. GLO, glucolacto-oligosaccharide; ITT, intention to treat; SAS, safety analysis set; FAS, full analysis set; PPS, per-protocol set. Safety was evaluated using ITT, as in SAS in this study; efficiency was assessed using PPS. Other reasons: excluded upon reaching the target sample size.

The study flow is shown in Figure 1. Healthy adult volunteers with a defecation frequency of five or fewer times per week were recruited through a volunteer bank. Of 191 applicants screened, 67 were excluded for not meeting the eligibility criteria (e.g., low Hb, high CK, and poor compliance), and 33 declined participation. Forty-one additional candidates who met the criteria were excluded because the target sample size had already been reached. Fifty participants were randomly allocated to the GLO or placebo group, and none withdrew during the four-week trial. One participant in the GLO group was excluded from the per-protocol set (PPS) analysis due to undisclosed habitual drinking, missing diary data, and repeated protocol violations. Therefore, data from 49 participants were included in the primary analysis. The baseline characteristics (Table 1; see Supplemental Table S3 for details) did not differ between the 49-participant group in the PPS and the 50-participant group in the full analysis set (FAS). No adverse events or factors possibly affecting defecation frequency or other secondary outcomes were confirmed through the defecation status questionnaire in FAS/PPS participants. No harmful events related to test food intake were reported in the safety analysis set (Figure 1).

### 3.2. Defecation Status

Defecation status is presented in Table 2, and changes in defecation frequency are illustrated in Supplemental Figure S6. The defecation frequency at 0 weeks and 1 week before intake was comparable between the two groups, ranging from 3 to 4 times per week. Although the defecation frequency of the placebo group increased slightly during the four-week period, that of the GLO group increased to a greater extent. The transition across the four time points in defecation frequency was significantly greater in the GLO group than in the placebo group. Along with the defecation frequency, the number of defecation days was significantly greater in the GLO group than in the placebo group. The average number of defecation days per week in the GLO group reached about 5 days per week after the intervention period. Stool volume and consistency were not significantly different between the two groups. However, in the GLO group, stool consistency tended to approach a score of 4 compared with the placebo group.

### 3.3. Relative Abundance of Fecal Microbiota

**Figure 2:**
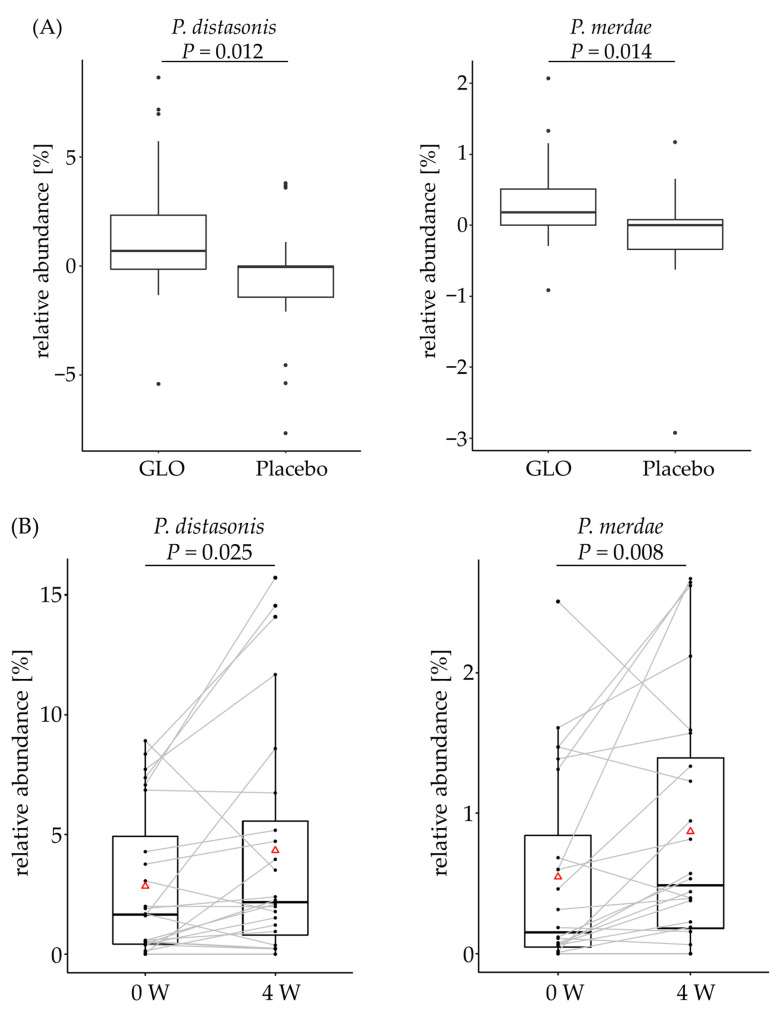
Species-level relative abundance in the genus of *Parabacteroides*. Species-level analysis was conducted using qPCR. Data are presented in a box plot (*n* = 24 for the GLO group and *n* = 25 for the placebo group). (**A**) Change rates of the relative abundance before and after test food intake between the GLO and placebo groups. *p* value versus placebo (Mann–Whitney U test). (**B**) Relative abundance before and after test food intake. Open triangles represent the mean value. Gray lines connect the paired data points for each participant. *p* value versus zero weeks (Wilcoxon signed-rank test). GLO, glucolacto-oligosaccharide.

Among the 189 bacterial taxa analyzed, Table 3 summarizes the relative abundances of the fecal microbiota and the changes in those that were significantly different between the two groups. Changes in the relative abundances of *Bacteroides*, *Parabacteroides*, *Phascolarctobacterium*, *Parasutterella*, and *Lachnospiraceae_UCG-010* were significantly higher in the GLO group than in the placebo group, although Bacteroides at 4 weeks did not increase in the GLO group compared with that at 0 weeks. Changes in the relative abundance of *Bifidobacterium* were significantly lower in the GLO group. Detailed species-level analysis using qPCR demonstrated that the change rates of the relative abundances of both *Parabacteroides distasonis* and *Parabacteroides merdae* were significantly higher in the GLO group than in the placebo group (Figure 2A). Additionally, the relative abundances of both bacteria at 4 weeks increased in the GLO group compared with those at 0 weeks (Figure 2B). The genus *Parabacteroides* was detected in 48 out of 50 subjects (96%).

### 3.4. Composition of Fecal Bile Acid

The relative abundances of the 10 analyzed primary and secondary fecal bile acids are summarized in Table 4. The GLO group showed a significant increase in the relative abundance of chenodeoxycholic acid (CDCA) and a significant decrease in the relative abundance of deoxycholic acid (DCA) compared with the placebo group. Additionally, isoallolithocholic acid (isoalloLCA) concentrations were significantly higher at 4 weeks than at baseline in the GLO group. No other bile acids showed significant differences either between or within groups.

### 3.5. Alpha Diversity Indices

Several alpha diversity indices with various characteristics are listed in Table 5. Changes in the Simpson diversity index and Pielou’s evenness index were significantly higher in the GLO group than in the placebo group. The actual values of these indices at 4 weeks also increased only in the GLO group compared with those at 0 weeks. Similarly, changes in the Shannon index tended to be higher in the GLO group than in the placebo group. The actual value of this index tended to be higher in the GLO group.

## 4. Discussion

This is the first human intervention study demonstrating that GLO improves defecation status and selectively increase *Parabacteroides*. As commonly demonstrated, 4-GK is a unique oligosaccharide that can increase both *Parabacteroides* and *Bifidobacterium* in monocultures in in vitro experiments [21]. The detailed evaluations in this study demonstrated that both 4-GK and GGK increased several species of *Parabacteroides* and *Bifidobacterium* in in vitro monocultures (see Supplemental Figure S2 for details). The present study suggests that GLO can reach the human colon and influence the gut microbiome. However, differences were observed between the previous human study and the present human study. When GLO was administered to humans, the previous study observed an increase in *Bifidobacterium* but not in *Parabacteroides* [21], whereas the present study observed an increase in *Parabacteroides* but not in *Bifidobacterium*. Although *Bifidobacterium* and *Parabacteroides* may have an antagonistic relationship [33], these discrepancies likely reflect variations in baseline gut microbiota composition and intervention materials. The previous study targeted healthy individuals without specific abdominal complaints. Individuals with low defecation frequency exhibit differences in the relative abundance of certain gut microbiota compared with the general population [34]. Differences in the target population may explain why *Parabacteroides* was selectively increased by GLO in individuals with a lower defecation frequency. Moreover, the previous human study used GLO syrup, which is a solution containing GLO, lactose, and fructose produced by the dextransucrase reaction of lactose and sucrose, rather than purified GLO [21]. Since differences in the test substances may have influenced the results, further accumulation of evidence is important.

In addition to 16S rRNA analysis, qPCR results confirmed significant increases in both *P. merdae* and *P. distasonis* in the GLO group. *P. merdae*, associated with supercentenarians, produces isoalloLCA, a metabolite linked to longevity [10]. Accordingly, the relative abundance of isoalloLCA rose significantly in the GLO group. Furthermore, predictive functional gene analysis revealed an increase in the relative abundance of 5α-reductase (EC 1.3.1.22), an enzyme required for isoalloLCA metabolism and known to be harbored by *P. merdae* [10]. The inferred increase in 5α-reductase was positively correlated with the increased abundance of *Parabacteroides* observed in the GLO group (see Supplemental Figure S4 for details). These results support the possibility that GLO may be related to longevity by enhancing isoalloLCA production by *P. merdae*. *P. distasonis* is also known as a beneficial species, as its reduced abundance has been observed in patients with multiple sclerosis. It has been reported to alleviate inflammatory arthritis and obesity and suppress non-alcoholic steatohepatitis (NASH) in in vivo models [35]. Our additional analysis also noted that the increase in *Parabacteroides* with GLO intake tended to show a negative correlation with γ-GT and TG levels, providing supporting evidence for this association (see Supplemental Figure S5 for details). Taken together with the observed increase in *P. distasonis*, the protective effects of GLO against metabolic, inflammatory, and hepatic diseases warrant further research.

Prebiotics are known to benefit human health by being catabolized by microbiota and modulating the gut environment. GLO is prebiotics; however, they are distinguished from other prebiotics by their unique capacity to selectively increase *Parabacteroides*, as mentioned above. To assess the actual benefits of increasing *Parabacteroides*, defecation frequency, an indicator of gut health, was evaluated as the primary outcome. In this randomized, double-blind, placebo-controlled clinical study, a four-week intervention with GLO significantly improved defecation frequency in individuals with lower defecation frequency (Table 2). The number of defecation days also improved significantly in the GLO group compared with that in the placebo group. A plausible mechanism of action underlying the increase in defecation frequency observed in the GLO group is a compositional change in bile acids driven by *Parabacteroides*. Bile acids are produced in the liver and enter the gut. Most bile acids are reabsorbed in the small intestine, whereas the remainder are detected in feces. Modification of bile acids by the gut microbiota can be broadly classified into two pathways. The first is the non-12-OH pathway, in which chenodeoxycholic acid (CDCA) is converted into ursodeoxycholic acid (UDCA) or lithocholic acid (LCA). *Parabacteroides* is a key producer of LCA and UDCA, and LCA, an FXR ligand, suppresses the cholic acid (CA)-producing pathway [36]. The second pathway is the 12-OH pathway, in which CA is converted to deoxycholic acid (DCA). DCA is a major bile acid that is mostly reabsorbed in the small intestine. Compared with DCA, bile acids such as CDCA tend to be excreted into the colon, where they can serve as a chemical stimulus for gut motility. The relative abundance of DCA in the GLO group significantly decreased in this study, suggesting that the total bile acids reaching the colon increased. Moreover, CDCA is known to promote gut motility [37], and our results showed a significant increase in CDCA in the GLO group compared with the placebo group (Table 4). Thus, these findings suggest that the increased defecation frequency observed in the GLO group may be associated with enhanced gut motility triggered by a greater delivery of bile acids to the colon. Elevated levels of *Parabacteroides* may contribute to this process. Notably, UDCA is used as a medication for hepatobiliary diseases, whereas DCA poses a risk of colon cancer. Therefore, compositional changes in the bile acids themselves, mediated by *Parabacteroides*, are significant for human health. In addition, the stool consistency score in the GLO group tended to increase compared with that in the placebo group, approaching a value of 4. Since a stool consistency score closer to 4 indicates normal stool consistency and a lower score indicates a harder stool [38], GLO may help soften and improve stool consistency. Therefore, GLO showed overall improvement in the defecation status of individuals with a lower defecation frequency.

To further assess other aspects of gut health, microbiota composition and alpha diversity were also evaluated. As presented in Table 5, multiple alpha diversity indices were increased in the GLO group relative to the placebo group, providing preliminary evidence of an intervention effect. The gut microbiota of centenarians tends to exhibit higher diversity, and alpha diversity has been reported as a useful predictor of longevity [39]. Additionally, alpha diversity is lower in individuals with diseases such as obesity, type 2 diabetes, and Crohn’s disease [40], suggesting that increased diversity may be a key factor for health and longevity. Among the various alpha diversity indices, Pielou’s evenness index is unique for measuring evenness. Our results suggest that not only did the number of constituent bacterial species increase, but the bacterial community was also balanced by attenuating the proportions of major bacterial taxa to sufficient levels. Compared with the Shannon index, the Simpson diversity index more strongly reflects the influence of the dominant species. Therefore, the difference observed in the Simpson index suggests that GLO helps balance the bacterial community; however, further investigation is required to elucidate the significance of balancing the gut microbiota.

In the present study, we placed particular emphasis on *Parabacteroides* because we observed an increase in its abundance [21]. Regarding the observed increase in the relative abundances of *Phascolarctobacterium*, *Parasutterella*, and *Lachnospiraceae*_*UCG-010* (Table 3), we considered *Parabacteroides* to be a potential key coordinator of the microbiota. The relationship between a decrease in *Bifidobacterium* and an increase in *Parabacteroides* and *Phascolarctobacterium* has been demonstrated in other studies [33]. Importantly, since GLO preferentially increases *Parabacteroides*, it is likely that *Parabacteroides* acts as the initiator of the increase in *Phascolarctobacterium* and decrease in *Bifidobacterium* in GLO intervention studies. Although a causal relationship was not elucidated, we believe that *Parabacteroides* may serve as the central coordinator of increased diversity in the microbiota. Supporting this hypothesis, our additional analysis also revealed a significant positive correlation between the GLO intake-induced increase in *Parabacteroides* and the GLO intake-induced increase in alpha diversity indices (see Supplemental Figure S3A for details). Furthermore, the baseline relative abundance of *Parabacteroides* was significantly positively correlated with several alpha diversity indices (see Supplemental Figure S3B for details). In the future, we would also like to examine other low-abundance bacteria that increased in response to GLO, provided that their reproducibility can be established.

This study has some limitations. First, the relative abundance of fecal bile acids was evaluated because their absolute concentrations could not be assessed. The absolute volume of total bile acids produced in the body depends on the composition and quantity of the diet [41]. Therefore, it is necessary to use standardized diets to evaluate their absolute concentrations. However, standardized diets were not used in this study to prioritize the evaluation of defecation frequency under natural dietary habits and reduce the burden on participants. Furthermore, as a common finding in the present results, elucidation of the causal relationship of GLO remains a subject for future investigation.

## 5. Conclusions

Our findings suggest that regular GLO intake improves gut health, as evidenced by improved defecation status, increased gut microbiota diversity, changes in bile acid composition, and a selective increase in *Parabacteroides*. The increase in *Parabacteroides* observed in this study was driven, at least in part, by an increase in *P. distasonis* and *P. merdae.* These increases suggest that GLO intake may confer further benefits, including for longevity, hepatic function, and lipid metabolism. Thus, GLO may represent novel upcycled prebiotics for promoting better digestion and healthy aging.

## Figures and Tables

**Table 1:** Baseline characteristics of study participants in the GLO and placebo groups in the PPS.

Characteristics	GLO	Placebo	*p* Value
**Number of participants**	24	25	
**Sex (female/male)**	12/12	12/13	1.000
**Age (years)** **[range]**	49.8 ± 9.6	49.2 ± 9.6	0.818
	[31–64]	[27–64]	
**BMI (kg/m^2^)**	23.1 ± 2.7	21.6 ± 2.5	0.039
**Defecation frequency (times/week)**	3.2 ± 0.5	3.1 ± 0.7	0.858

Data are shown as mean ± standard deviation. Between-group (GLO and placebo) comparisons were performed using Fisher’s exact test for sex and an unpaired *t*-test for age, BMI, and defecation frequency. GLO, glucolacto-oligosaccharide.

**Table 2:** Defecation status in the GLO and placebo groups during the pre-intake and 4-week intake periods.

Items	Group	0 Weeks	1 Week	2 Weeks	3 Weeks	4 Weeks	*p* Value
**Defecation** **frequency** **(times/week)**	GLO	3.2 ± 0.2	4.3 ± 0.3	5.4 ± 0.4	5.5 ± 0.5	5.8 ± 0.6	0.029
	Placebo	3.4 ± 0.3	4.2 ± 0.3	4.1 ± 0.3	4.6 ± 0.3	4.4 ± 0.3	
**Number of** **defecation days** **(days/week)**	GLO	3.1 ± 0.2	4.0 ± 0.2	4.8 ± 0.3	4.8 ± 0.3	5.0 ± 0.3	0.024
	Placebo	3.2 ± 0.2	3.8 ± 0.2	3.8 ± 0.2	4.0 ± 0.3	4.2 ± 0.3	
**Stool volume** **(unit/weeks)**	GLO	2.8 ± 0.3	2.7 ± 0.2	2.6 ± 0.2	2.7 ± 0.2	2.7 ± 0.3	0.622
	Placebo	2.3 ± 0.3	2.2 ± 0.2	2.2 ± 0.2	2.3 ± 0.2	2.2 ± 0.2	
**Stool** **consistency** **(score/week)**	GLO	3.5 ± 0.1	3.8 ± 0.2	4.0 ± 0.2	3.9 ± 0.1	4.0 ± 0.2	0.063
	Placebo	3.6 ± 0.2	3.8 ± 0.2	3.6 ± 0.2	3.4 ± 0.2	3.4 ± 0.2	

Data are presented as mean ± standard error (*n* = 24 for the GLO group and *n* = 25 for the placebo group). Between-group comparisons were performed using a mixed-effects model for repeated measures. Stool volume is calculated with the volume of a medium-sized chicken egg defined as one unit. Stool consistency is scored using the Bristol Stool Scale, where lower scores indicate harder stools and higher scores indicate softer stools. GLO, glucolacto-oligosaccharide.

**Table 3:** Relative abundance of bacterial genus in the GLO and placebo groups during the pre-intake and 4-week intake periods.

Genus	Group	0 Weeks	4 Weeks	*p* Value	Δ 4 − 0 Weeks	*p* Value
***Bifidobacterium***	GLO	13.6 ± 2.5	9.7 ± 2.1	0.000	−3.9 ± 1.1	0.038
	Placebo	13.9 ± 1.9	14.3 ± 2.3	0.842	0.5 ± 1.5	
***Bacteroides***	GLO	5.6 ± 0.8	7.9 ± 1.2	0.077	2.3 ± 1.0	0.009
	Placebo	8.3 ± 1.4	6.4 ± 0.9	0.083	−1.9 ± 1.1	
***Parabacteroides***	GLO	0.8 ± 0.2	1.3 ± 0.2	0.010	0.5 ± 0.2	0.006
	Placebo	0.8 ± 0.1	0.7 ± 0.1	0.316	−0.1 ± 0.1	
***Phascolarctobacterium***	GLO	0.4 ± 0.2	0.7 ± 0.2	0.036	0.2 ± 0.1	0.000
	Placebo	0.8 ± 0.2	0.4 ± 0.1	0.002	−0.4 ± 0.1	
***Parasutterella***	GLO	0.1 ± 0.0	0.3 ± 0.1	0.010	0.2 ± 0.1	0.003
	Placebo	0.2 ± 0.1	0.1 ± 0.1	0.050	−0.1 ± 0.1	
***Lachnospiraceae*** ***UCG-010***	GLO	0.0 ± 0.0	0.1 ± 0.0	0.028	0.1 ± 0.0	0.004
	Placebo	0.0 ± 0.0	0.0 ± 0.0	0.345	0.0 ± 0.0	

NGS-based 16S rRNA analysis was conducted, and bacterial genera showing statistically significant differences between the groups are summarized in this table. Data are presented as mean ± standard error (*n* = 24 for the GLO group and *n* = 25 for the placebo group). Between-group comparisons were analyzed using the Mann–Whitney U test, and between-period comparisons were analyzed using the Wilcoxon signed-rank test.

**Table 4:** Comparison of fecal bile acid in the GLO and placebo groups before and after test food intake.

	Group	0 Weeks	4 Weeks	*p* Value	Δ 4 − 0 Weeks	*p* Value
**OxoLCA**	GLO	1.0 ± 0.3	1.2 ± 0.4	0.915	0.1 ± 0.2	0.316
	Placebo	1.3 ± 0.2	1.0 ± 0.2	0.230	−0.3 ± 0.2	
**IsoalloLCA**	GLO	0.4 ± 0.2	1.0 ± 0.6	0.042	0.6 ± 0.5	0.148
	Placebo	0.3 ± 0.1	0.6 ± 0.2	0.916	0.2 ± 0.2	
**IsoLCA**	GLO	5.6 ± 1.0	4.5 ± 0.9	0.546	−1.1 ± 1.1	0.345
	Placebo	8.4 ± 1.2	8.7 ± 1.3	0.615	0.3 ± 1.2	
**LCA**	GLO	20.2 ± 3.1	16.9 ± 3.2	0.509	−3.4 ± 2.8	0.804
	Placebo	25.9 ± 2.7	24.1 ± 2.4	0.508	−1.8 ± 2.0	
**UDCA**	GLO	3.0 ± 0.9	3.9 ± 1.2	0.345	0.9 ± 1.0	0.545
	Placebo	1.5 ± 0.6	1.7 ± 0.6	0.711	0.2 ± 0.5	
**HDCA**	GLO	0.2 ± 0.0	0.2 ± 0.1	0.157	0.0 ± 0.0	0.270
	Placebo	0.2 ± 0.0	0.2 ± 0.0	0.833	0.0 ± 0.0	
**CDCA**	GLO	6.8 ± 2.0	9.6 ± 2.3	0.208	2.9 ± 2.6	0.036
	Placebo	5.1 ± 1.9	3.6 ± 1.5	0.042	−1.5 ± 0.9	
**DCA**	GLO	39.8 ± 4.7	31.3 ± 4.4	0.053	−8.5 ± 4.9	0.004
	Placebo	42.6 ± 4.0	49.8 ± 4.0	0.052	7.1 ± 3.6	
**UCA**	GLO	5.2 ± 1.9	10.4 ± 3.3	0.178	5.1 ± 3.6	0.127
	Placebo	1.4 ± 0.7	1.3 ± 0.5	0.300	0.0 ± 0.2	
**CA**	GLO	16.7 ± 5.4	18.6 ± 4.3	0.546	1.9 ± 4.2	0.166
	Placebo	12.4 ± 5.0	7.8 ± 4.0	0.191	−4.5 ± 3.2	

Data are presented as mean ± standard error (*n* = 24 for the GLO group and *n* = 25 for the placebo group). Between-group comparisons were analyzed using the Mann–Whitney U test, and between-period comparisons were analyzed using the Wilcoxon signed-rank test. OxoLCA, 3-oxo-lithocholic acid; IsoalloLCA, isoallolithocholic acid; IsoLCA, isolithocholic acid; LCA, lithocholic acid; UDCA, ursodeoxycholic acid; HDCA, hyodeoxycholic acid; CDCA, chenodeoxycholic acid; DCA, deoxycholic acid; UCA, ursocholic acid; CA, cholic acid; GLO, glucolacto-oligosaccharide.

**Table 5:** Changes in alpha diversity indices in the GLO and placebo groups before and after test food intake.

	Group	0 Weeks	4 Weeks	*p* Value	Δ 4 − 0 Weeks	*p* Value
**Simpson**	GLO	0.947 ± 0.005	0.954 ± 0.004	0.020	0.006 ± 0.003	0.041
	Placebo	0.954 ± 0.003	0.951 ± 0.004	0.408	−0.003 ± 0.004	
**Shannon**	GLO	5.347 ± 0.099	5.474 ± 0.092	0.055	0.127 ± 0.063	0.074
	Placebo	5.483 ± 0.088	5.431 ± 0.078	0.492	−0.053 ± 0.075	
**Pielou** **’s**	GLO	0.762 ± 0.008	0.773 ± 0.007	0.048	0.011 ± 0.005	0.022
	Placebo	0.769 ± 0.007	0.758 ± 0.007	0.152	−0.011 ± 0.008	

Data are presented as mean ± standard error (*n* = 24 for the GLO group and *n* = 25 for the placebo group). Between-group comparisons were analyzed using an unpaired *t*-test, and between-period comparisons were analyzed using a paired *t*-test. GLO, glucolacto-oligosaccharide.

## Data Availability

Data supporting the results of this study are available from the corresponding author upon reasonable request. However, data from the test food analysis are not publicly available because they include confidential product specifications.

## Supplementary Materials

The following supporting information can be downloaded at: https://www.mdpi.com/article/10.3390/microorganisms14050955/s1, Supplemental Table S1: Ingredients of test foods; Supplemental Table S2: Primer sequences and PCR conditions for target bacteria; Supplemental Table S3: Baseline characteristics in the FAS; Supplemental Figure S1: Definition of the name of glucolacto-oligosaccharide; Supplemental Figure S2: Turbidity of the bacterial monoculture with the addition of 4 GK or GGK; Supplemental Figure S3: Correlations between *Parabacteroides* and alpha diversity indices; Supplemental Figure S4: Analysis of PICRUSt2-predicted 5α-reductase abundance and its associations. Supplemental Figure S5: Correlations between changes in the relative abundances of *Parabacteroides*, TG, and γ-GT during GLO intake. Supplemental Figure S6: Transition across four time points in changes of defecation frequency.

## Abbreviations

The following abbreviations are used in this manuscript:

CA	Cholic acid
CDCA	Chenodeoxycholic acid
DCA	Deoxycholic acid
FAS	Full analysis set
γ-GT	Gamma-glutamyltransferase
GLO	Glucolacto-oligosaccharide
LCA	Lithocholic acid
NASH	Non-alcoholic steatohepatitis
PPS	Per-protocol set
TG	Triglyceride

## References

[1] Gupta V.K., Paul S., Dutta C. (2017). Geography, Ethnicity or Subsistence-Specific Variations in Human Microbiome Composition and Diversity. Front. Microbiol..

[2] Senghor B., Sokhna C., Ruimy R., Lagier J.-C. (2018). Gut microbiota diversity according to dietary habits and geographical provenance. Hum. Microbiome J..

[3] Rosenberg E. (2024). Diversity of bacteria within the human gut and its contribution to the functional unity of holobionts. npj Biofilms Microbiomes.

[4] Cekanaviciute E., Yoo B.B., Runia T.F., Debelius J.W., Singh S., Nelson C.A., Kanner R., Bencosme Y., Lee Y.K., Hauser S.L. (2017). Gut bacteria from multiple sclerosis patients modulate human T cells and exacerbate symptoms in mouse models. Proc. Natl. Acad. Sci. USA.

[5] Del Chierico F., Nobili V., Vernocchi P., Russo A., De Stefanis C., Gnani D., Furlanello C., Zandona A., Paci P., Capuani G. (2017). Gut microbiota profiling of pediatric nonalcoholic fatty liver disease and obese patients unveiled by an integrated meta-omics-based approach. Hepatology.

[6] Chen Y., Jiang S., Wang H., Si M., Wu H., Liang X., Yao S., Zhang Y., Wen X., Yang J. (2025). The Probiotic Parabacteroides johnsonii Ameliorates Metabolic Disorders Through Promoting BCAAs to BSCFAs Conversion. Adv. Sci..

[7] Haro C., Garcia-Carpintero S., Alcala-Diaz J.F., Gomez-Delgado F., Delgado-Lista J., Perez-Martinez P., Rangel Zuniga O.A., Quintana-Navarro G.M., Landa B.B., Clemente J.C. (2016). The gut microbial community in metabolic syndrome patients is modified by diet. J. Nutr. Biochem..

[8] Cui Y., Zhang L., Wang X., Yi Y., Shan Y., Liu B., Zhou Y., Lu X. (2022). Roles of intestinal Parabacteroides in human health and diseases. FEMS Microbiol. Lett..

[9] Fujiwara S., Park J., Takeda M., Miyatake T., Saito Y., Makino S., Kim Y.G. (2026). Sialic acid-responsive Parabacteroides is linked to gut barrier integrity in older adults. Gut Microbes.

[10] Sato Y., Atarashi K., Plichta D.R., Arai Y., Sasajima S., Kearney S.M., Suda W., Takeshita K., Sasaki T., Okamoto S. (2021). Novel bile acid biosynthetic pathways are enriched in the microbiome of centenarians. Nature.

[11] Park J., Kato K., Murakami H., Hosomi K., Tanisawa K., Nakagata T., Ohno H., Konishi K., Kawashima H., Chen Y.A. (2021). Comprehensive analysis of gut microbiota of a healthy population and covariates affecting microbial variation in two large Japanese cohorts. BMC Microbiol..

[12] Gibson G.R., Probert H.M., Loo J.V., Rastall R.A., Roberfroid M.B. (2004). Dietary modulation of the human colonic microbiota: Updating the concept of prebiotics. Nutr. Res. Rev..

[13] Gibson G.R., Hutkins R., Sanders M.E., Prescott S.L., Reimer R.A., Salminen S.J., Scott K., Stanton C., Swanson K.S., Cani P.D. (2017). Expert consensus document: The International Scientific Association for Probiotics and Prebiotics (ISAPP) consensus statement on the definition and scope of prebiotics. Nat. Rev. Gastroenterol. Hepatol..

[14] Davani-Davari D., Negahdaripour M., Karimzadeh I., Seifan M., Mohkam M., Masoumi S.J., Berenjian A., Ghasemi Y. (2019). Prebiotics: Definition, Types, Sources, Mechanisms, and Clinical Applications. Foods.

[15] Sakai Y., Seki N., Hamano K., Ochi H., Abe F., Masuda K., Iino H. (2019). Prebiotic effect of two grams of lactulose in healthy Japanese women: A randomised, double-blind, placebo-controlled crossover trial. Benef. Microbes.

[16] Arioz Tunc H., Calder P.C., Cait A., Dodd G.F., Gasaly Retamal N.Y.I., Guillemet D., James D., Korzeniowski K.J., Lubkowska A., Meynier A. (2025). Impact of non-digestible carbohydrates and prebiotics on immunity, infections, inflammation and vaccine responses: A systematic review of evidence in healthy humans and a discussion of mechanistic proposals. Crit. Rev. Food Sci. Nutr..

[17] Smolinska S., Popescu F.D., Zemelka-Wiacek M. (2025). A Review of the Influence of Prebiotics, Probiotics, Synbiotics, and Postbiotics on the Human Gut Microbiome and Intestinal Integrity. J. Clin. Med..

[18] Al-Habsi N., Al-Khalili M., Haque S.A., Elias M., Olqi N.A., Al Uraimi T. (2024). Health Benefits of Prebiotics, Probiotics, Synbiotics, and Postbiotics. Nutrients.

[19] Wang X., Yang J., Qiu X., Wen Q., Liu M., Zhou D., Chen Q. (2021). Probiotics, Pre-biotics and Synbiotics in the Treatment of Pre-diabetes: A Systematic Review of Randomized Controlled Trials. Front. Public Health.

[20] Wang L., Pan X., Jiang L., Chu Y., Gao S., Jiang X., Zhang Y., Chen Y., Luo S., Peng C. (2022). The Biological Activity Mechanism of Chlorogenic Acid and Its Applications in Food Industry: A Review. Front. Nutr..

[21] Negishi H., Ishida J., Ichikawa A., Asami I., Hagiwara S., Ohtake J., Hojo K., Kano H., Makino S. (2025). Lactose-Derived Glucolacto-Oligosaccharides Increase Specific Beneficial Bacteria in the Human Gut Microbiome. ACS Food Sci. Technol..

[22] Schmidt F.M., Santos V.L. (2014). Prevalence of constipation in the general adult population: An integrative review. J. Wound Ostomy Cont. Nurs..

[23] Kinoshita Y., Shoji S., Hayashi T., Okumura H. (2020). A cross-sectional analysis of the health-related quality of life and work productivity in Japanese subjects with self-reported chronic constipation using the National Health and Wellness Survey 2017. Nippon Shokakibyo Gakkai Zasshi.

[24] Yamakawa C., Kinoshita Y., Suzuki N., Takara T. (2022). Effects of Consuming a Test Beverage Containing Lactiplantibacillus plantarum SN13T Strain and Isomaltooligosaccharides on Bowel Movement and Intestinal Environment in Healthy Japanese People Aged ≥ 20 Years—A Randomized Double-blind Placebo-controlled Parallel-group Trial. Jpn. Pharmacol. Ther..

[25] Higuchi H., Takahashi H., Ando T., Yonejima Y., Iwama Y., Yoshida K. (2021). Effects of Chocolate Containing Lactobacillus brevis NTT001 and Galactooligosaccharides on Intestine—A Randomized, Double-blind, Placebo-controlled, Crossover Trial. Jpn. Pharmacol. Ther..

[26] Fukami K., Suehiro D., Ohnishi M. (2020). In Vitro Utilization Characteristics of Maltobionic Acid and Its Effects on Bowel Movements in Healthy Subjects. J. Appl. Glycosci..

[27] Takahashi S., Tomita J., Nishioka K., Hisada T., Nishijima M. (2014). Development of a prokaryotic universal primer for simultaneous analysis of Bacteria and Archaea using next-generation sequencing. PLoS ONE.

[28] Bolyen E., Rideout J.R., Dillon M.R., Bokulich N.A., Abnet C.C., Al-Ghalith G.A., Alexander H., Alm E.J., Arumugam M., Asnicar F. (2019). Reproducible, interactive, scalable and extensible microbiome data science using QIIME 2. Nat. Biotechnol..

[29] Callahan B.J., McMurdie P.J., Rosen M.J., Han A.W., Johnson A.J., Holmes S.P. (2016). DADA2: High-resolution sample inference from Illumina amplicon data. Nat. Methods.

[30] Quast C., Pruesse E., Yilmaz P., Gerken J., Schweer T., Yarza P., Peplies J., Glockner F.O. (2013). The SILVA ribosomal RNA gene database project: Improved data processing and web-based tools. Nucleic Acids Res..

[31] Douglas G.M., Maffei V.J., Zaneveld J.R., Yurgel S.N., Brown J.R., Taylor C.M., Huttenhower C., Langille M.G.I. (2020). PICRUSt2 for prediction of metagenome functions. Nat. Biotechnol..

[32] Hagio M., Matsumoto M., Fukushima M., Hara H., Ishizuka S. (2009). Improved analysis of bile acids in tissues and intestinal contents of rats using LC/ESI-MS. J. Lipid Res..

[33] Odamaki T., Kato K., Sugahara H., Hashikura N., Takahashi S., Xiao J.Z., Abe F., Osawa R. (2016). Age-related changes in gut microbiota composition from newborn to centenarian: A cross-sectional study. BMC Microbiol..

[34] Mancabelli L., Milani C., Lugli G.A., Turroni F., Mangifesta M., Viappiani A., Ticinesi A., Nouvenne A., Meschi T., van Sinderen D. (2017). Unveiling the gut microbiota composition and functionality associated with constipation through metagenomic analyses. Sci. Rep..

[35] Duan J., Li Q., Cheng Y., Zhu W., Liu H., Li F. (2024). Therapeutic potential of Parabacteroides distasonis in gastrointestinal and hepatic disease. MedComm.

[36] Goodwin B., Jones S.A., Price R.R., Watson M.A., McKee D.D., Moore L.B., Galardi C., Wilson J.G., Lewis M.C., Roth M.E. (2000). A regulatory cascade of the nuclear receptors FXR, SHP-1, and LRH-1 represses bile acid biosynthesis. Mol. Cell.

[37] Bampton P.A., Dinning P.G., Kennedy M.L., Lubowski D.Z., Cook I.J. (2002). The proximal colonic motor response to rectal mechanical and chemical stimulation. Am. J. Physiol.-Gastrointest. Liver Physiol..

[38] Sato S., Kawamura H., Ooya W. (1991). Acinic cell tumor of the hard palate. Int. J. Oral Maxillofac. Surg..

[39] Kong F., Hua Y., Zeng B., Ning R., Li Y., Zhao J. (2016). Gut microbiota signatures of longevity. Curr. Biol..

[40] Gupta V.K., Kim M., Bakshi U., Cunningham K.Y., Davis J.M., Lazaridis K.N., Nelson H., Chia N., Sung J. (2020). A predictive index for health status using species-level gut microbiome profiling. Nat. Commun..

[41] David L.A., Maurice C.F., Carmody R.N., Gootenberg D.B., Button J.E., Wolfe B.E., Ling A.V., Devlin A.S., Varma Y., Fischbach M.A. (2014). Diet rapidly and reproducibly alters the human gut microbiome. Nature.

