# Supplementary material for: Benefits of Regular Intake of Glucolacto-Oligosaccharides on Gut Health in Adults with Low Defecation Frequency: A Randomized, Double-Blind, Placebo-Controlled Study
Source: Microorganisms. 2026 Apr 23;14(5):955. doi: 10.3390/microorganisms14050955 (PMC13209567; doi:10.3390/microorganisms14050955)
Supplement: Supplementary file 1 [file microorganisms-14-00955-s001.zip › microorganisms-4207046-supplementary.pdf]

Supplemental Table S1. Ingredients of test foods

Ingredient	GLO (g/pack)	Placebo (g/pack)
Fructose	0.01	1.17
Glucose	0	0.85
Glucolacto-oligosaccharides	1.4	0
Citric acid	0.05	0.05
Sodium citrate	0.03	0.03

The placebo was sweetened with fructose and glucose. GLO, Glucolacto-oligosaccharides.

Supplemental Table S2. Primer sequences and PCR conditions for target bacteria

Target bacteria	Primer sequence (5′-3′)
Parabacteroides distasonis	F: AACACCTTTTCTAGCAATAGCCGT<br>R: CTAACCTGTTAGTAACTAGTGGCAAG
Parabacteroides merdae	F: CTAGGAGTTTGCGATACAATGTAAG<br>R: TTCCACTCCGGTCAGACTA
Total bacteria	F: ACTCCTACGGGAGGCAGCAGT<br>R: GTATTACCGCGGCTGCTGGCAC

Supplemental Table S3. Baseline characteristics of study participants in the GLO and the placebo groups in the FAS

**Characteristics**	**GLO**			**Placebo**			***P* value**
Number of participants		25			25		
Sex (Female/Male)	13	/	12	12	/	13	1.000
Age (years)	49.1	±	10.1	49.2	±	9.6	0.969
BMI (kg/m^2)	23.0	±	2.7	21.6	±	2.5	0.059
Defecation frequency (times/week)	3.2	±	0.5	3.1	±	0.7	0.706

Data are shown as mean ± standard deviation. Between-group (GLO and placebo) comparisons were performed using Fisher's exact test for sex, and an unpaired *t*-test for age, BMI and Defecation frequency. GLO, Glucolacto-oligosaccharides.

Generic name	Glucolacto-oligosaccharide (GLO)	
Substance name	4-galactosylkojibiose (4-GK)	Glucosyl-galactosylkojibiose (GGK)
Classification	Triose	Tetraose
Conformation	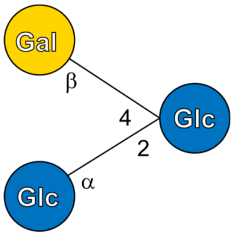	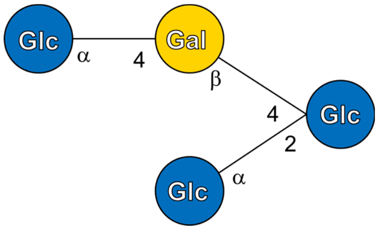

Supplemental Figure S1. Definition of the name of glucolacto-oligosaccharide. 4-GK consists of a structure in which one glucose molecule is transglycosylated onto lactose, while GGK has an additional glucose molecule attached to 4-GK.

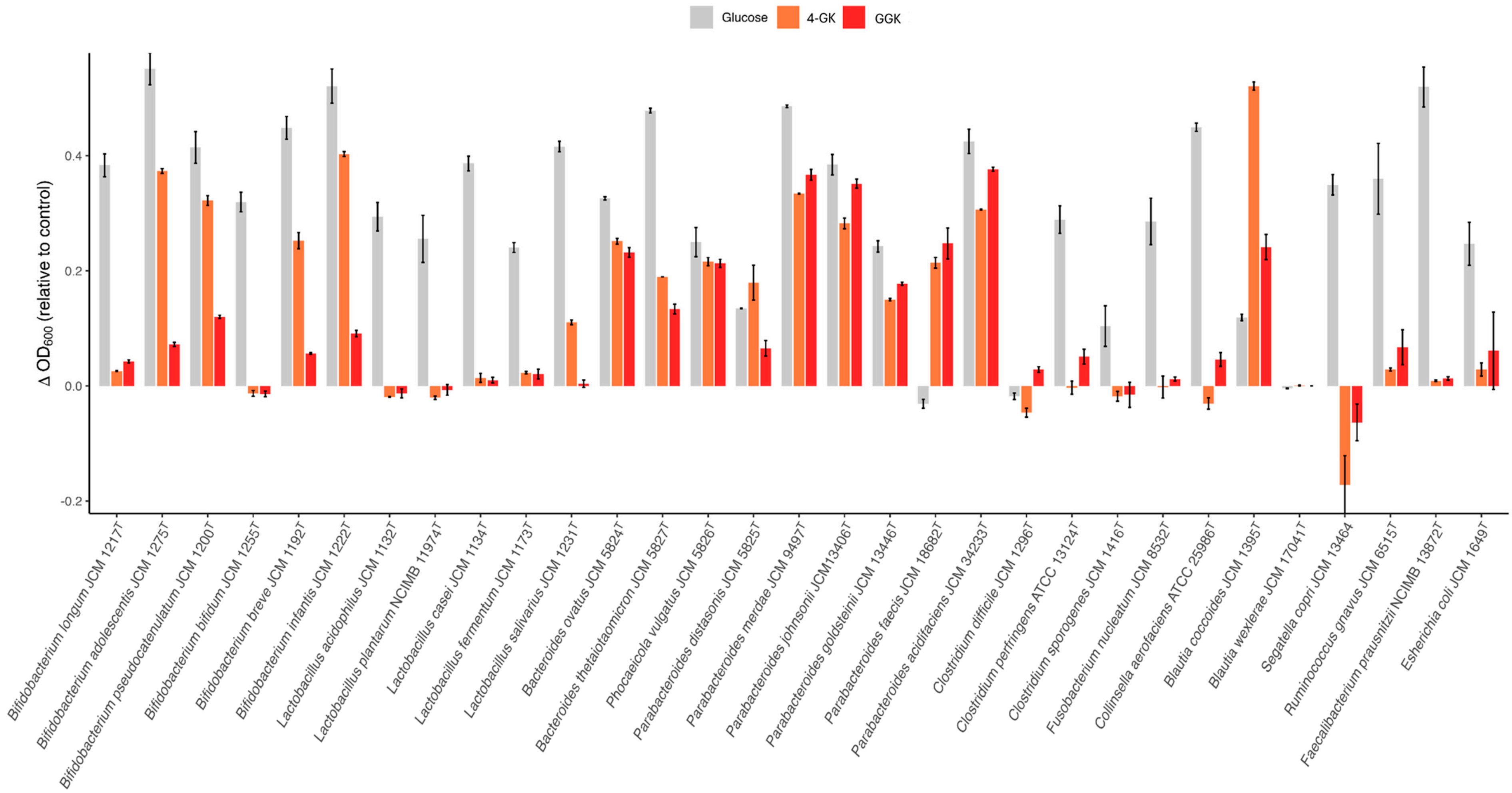

Supplemental Figure S2. Turbidity of the bacterial monoculture with the addition of 4GK or GGK. The graph shows the difference in turbidity between the blank and each sugar-added condition. The gray bars indicate results from monoculture of each bacteria with glucose, the orange bars with 4GK, and the red bars with GGK. 4-GK, 4-galactosylkojibiose; GGK, glucosyl-galactosylkojibiose.

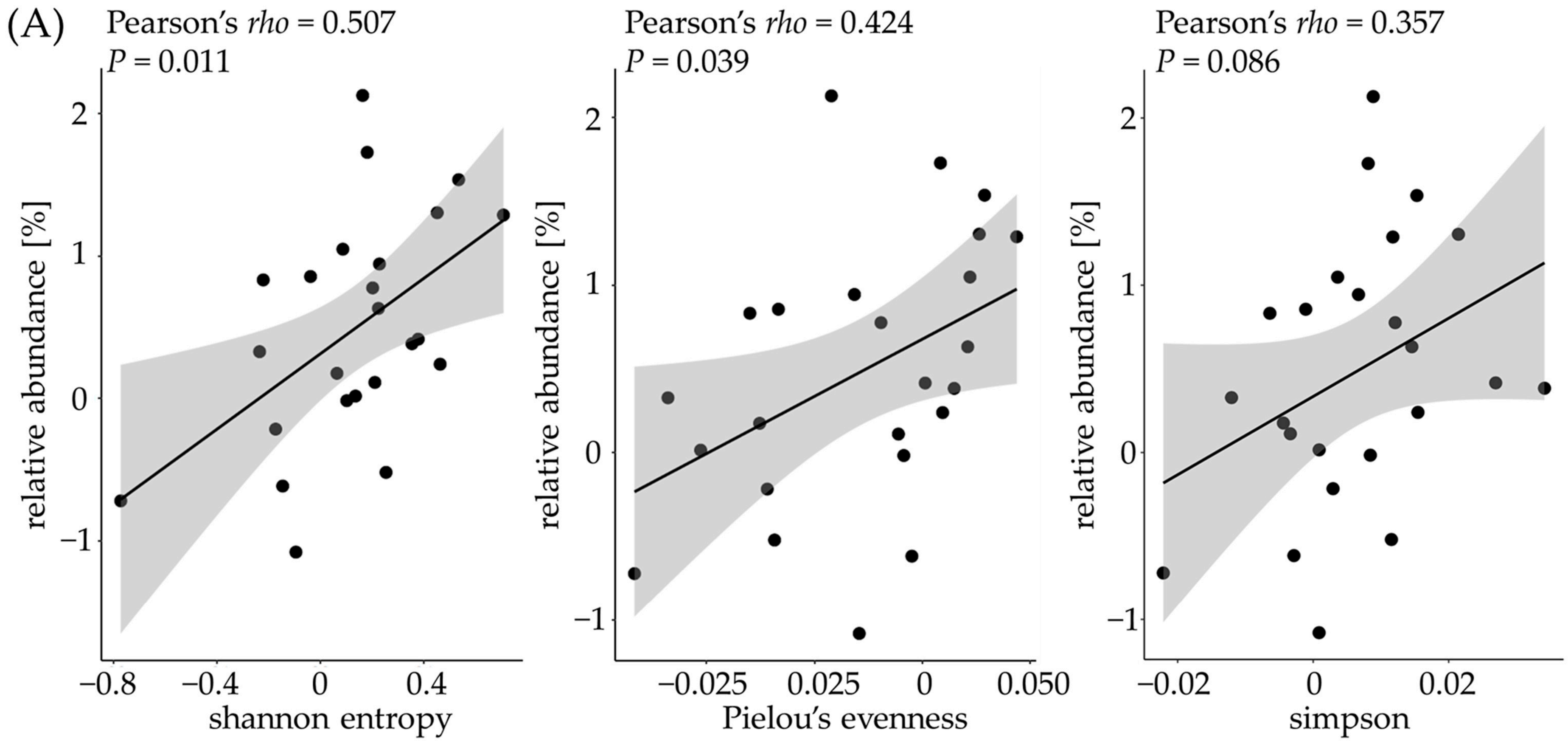

(A)
Pearson's *rho* = 0.507
P = 0.011
relative abundance [%]
shannon entropy
Pearson's *rho* = 0.424
P = 0.039
relative abundance [%]
Pielou's evenness
Pearson's *rho* = 0.357
P = 0.086
relative abundance [%]
simpson

(B)

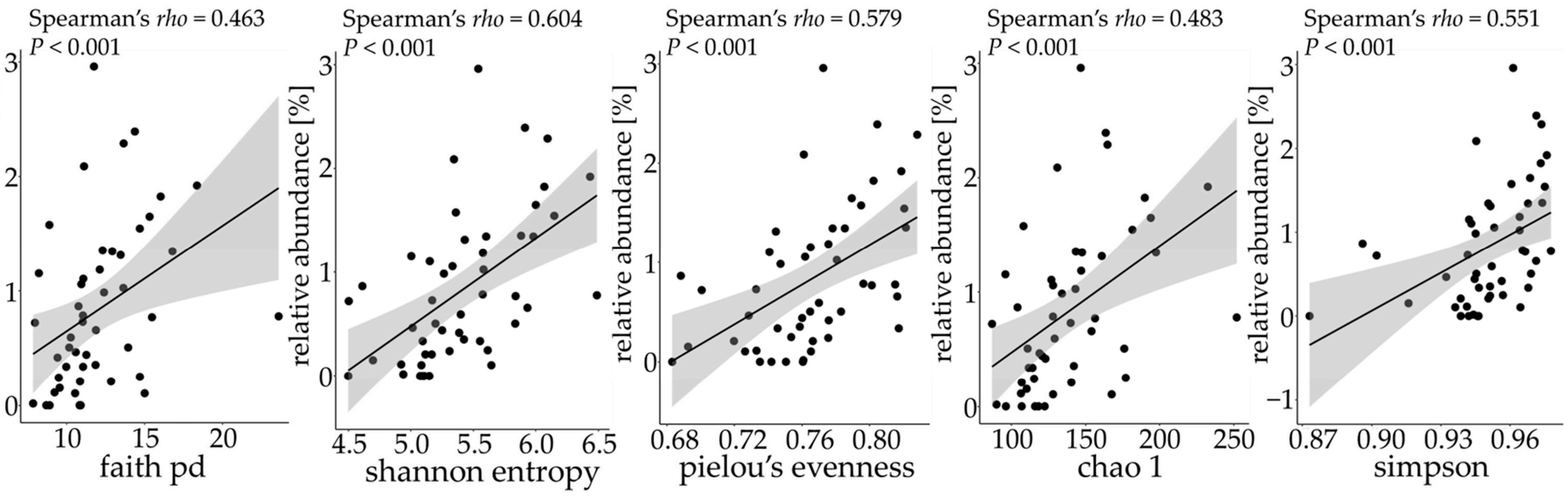

Supplemental Figure S3. Correlations between *Parabacteroides* and alpha diversity indices. (A) Correlation between changes in the relative abundance of *Parabacteroides* and alpha diversity indices during GLO intake. Spearman's correlation coefficients were analyzed (n = 24 in the GLO group). (B) Correlations between the basal relative abundance of *Parabacteroides* and alpha diversity indices in the GLO and placebo groups. Spearman's correlation coefficients were analyzed (n = 50 in the GLO and placebo groups). GLO, Glucolacto-oligosaccharides.

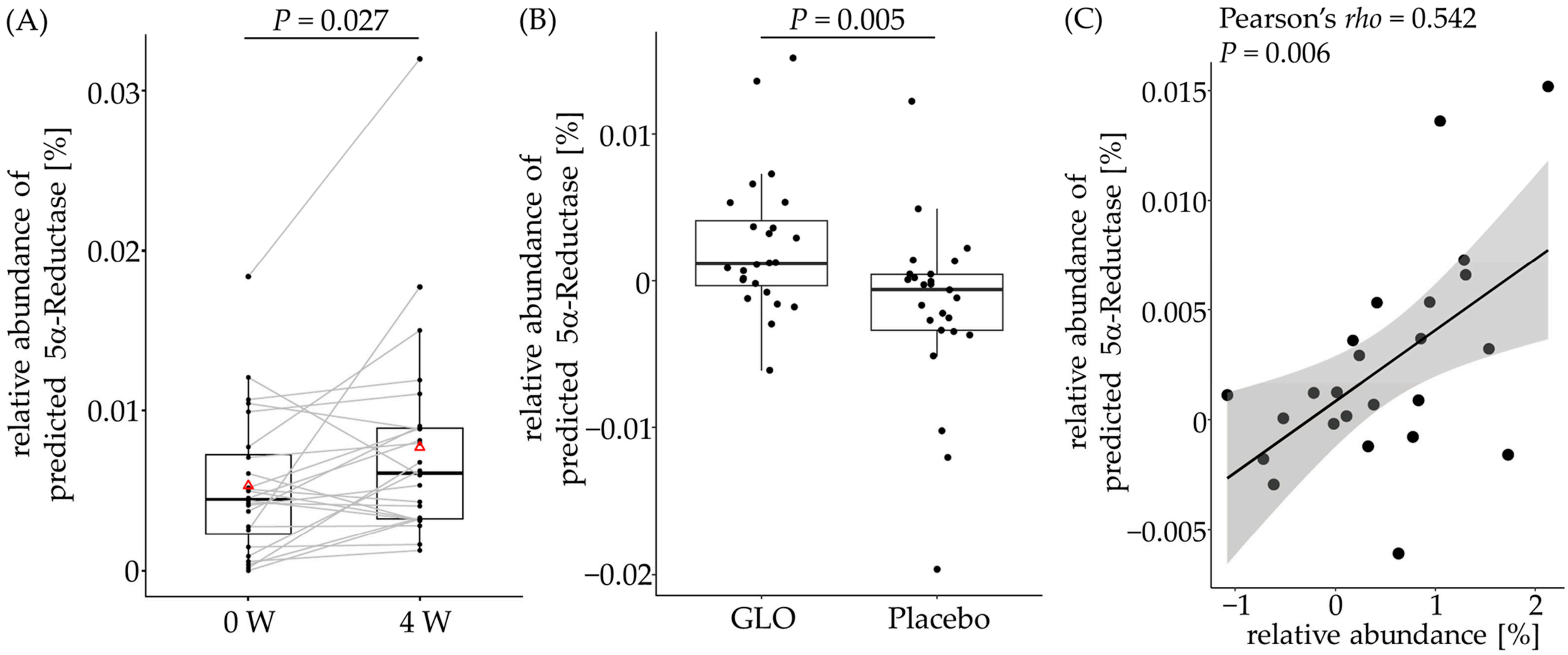

Supplemental Figure S4. (A) Comparison of the relative abundance of predicted 5α-reductase at 0 week and 4 week using the Wilcoxon signed-rank test (n = 24 in the GLO group). (B) Comparison of changes in the relative abundance of predicted 5α-Reductase between the GLO and the placebo groups using the Mann–Whitney U test (n = 24 for the GLO group and n = 25 for the placebo group). (C) Correlations between changes in the relative abundance of predicted 5α-reductase and *Parabacteroides* relative abundance. Pearson's correlation coefficients were analyzed (n = 24 in the GLO group). Open triangles represent the mean value. Gray lines connect the paired data points for each participant. GLO, Glucolacto-oligosaccharides.

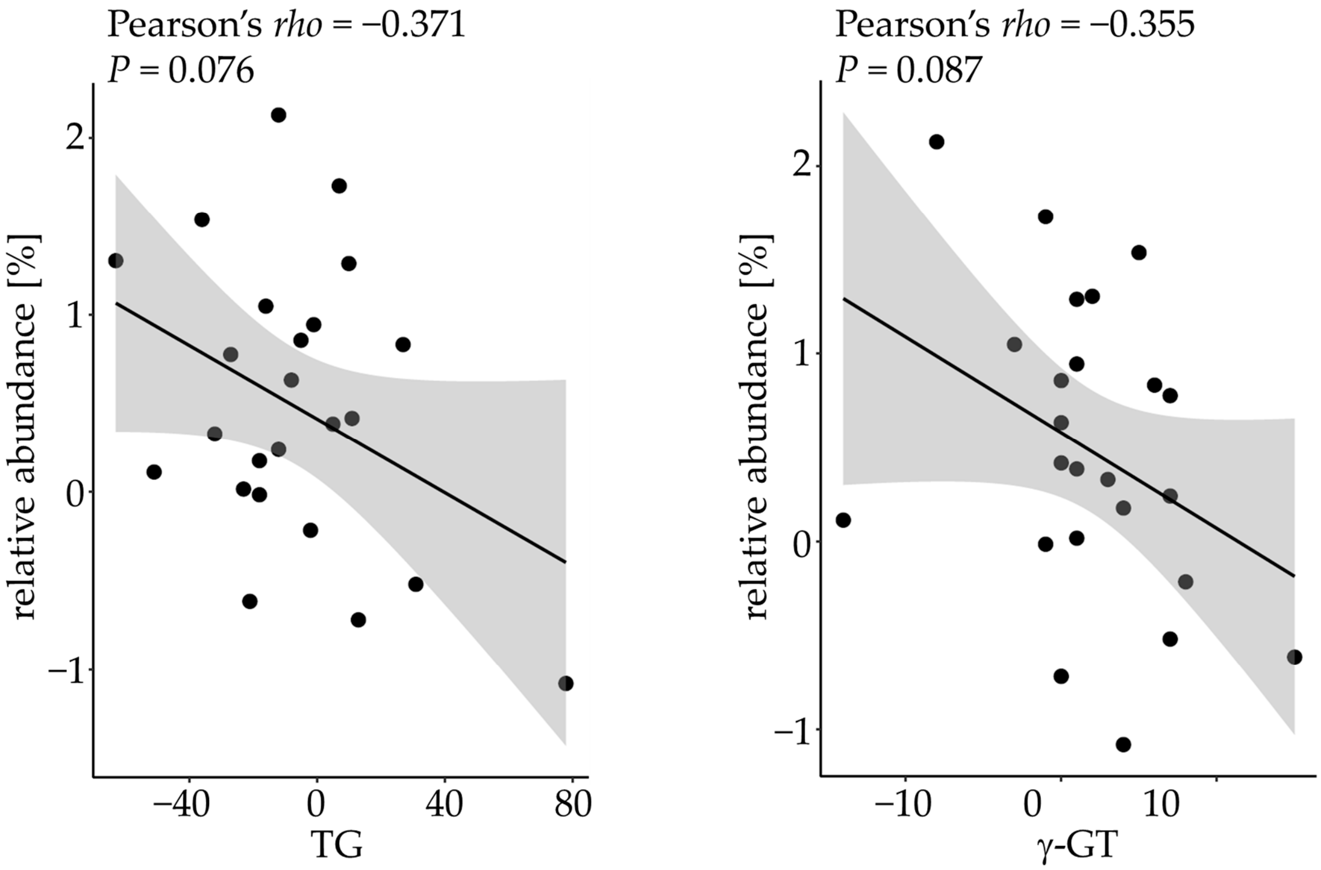

Supplemental Figure S5. Correlations between changes in the relative abundances of *Parabacteroides*, TG, and γ-GT during GLO intake. Pearson's correlation coefficients were analyzed (*n* = 24 in the GLO group). TG, triglyceride; γ-GT, gamma-glutamyltransferase; GLO, Glucolacto-oligosaccharides.

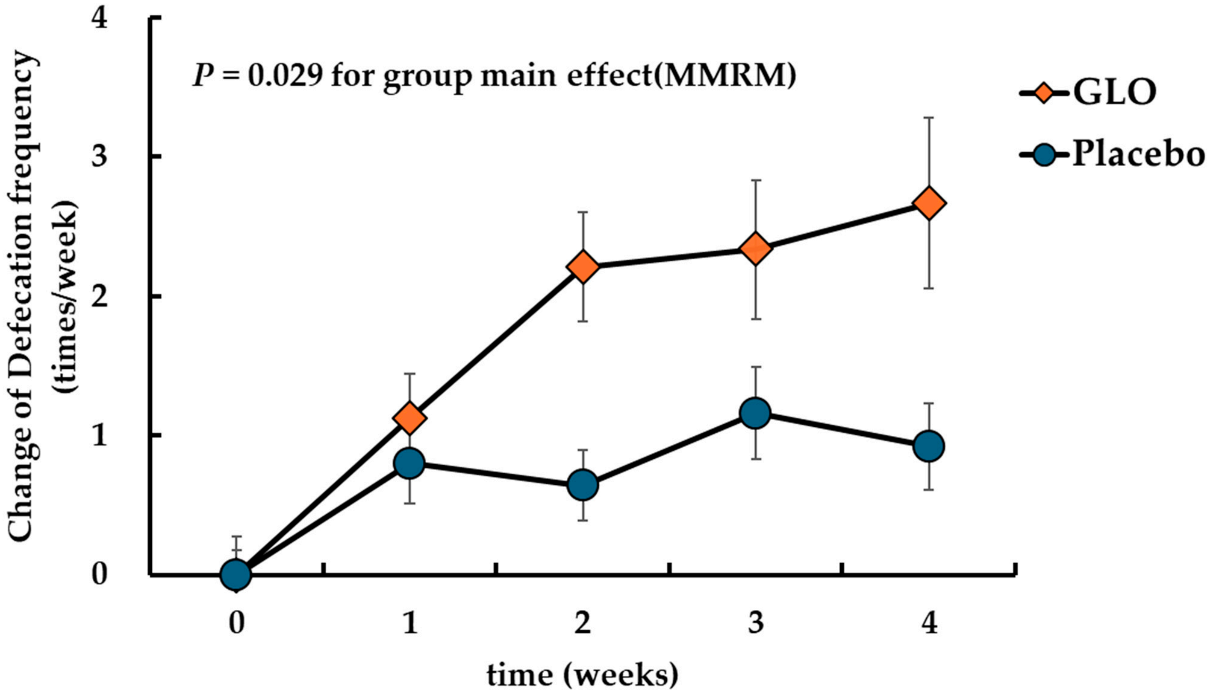

Supplemental Figure S6. Transition across four time points in changes of defecation frequency. Data are presented as mean ± standard error (*n* = 24 for the GLO group and *n* = 25 for the placebo group). Filled diamond represent GLO intake. Filled circles represent placebo intake. *P*-value versus placebo (Mixed-Effects Model for Repeated Measures). GLO, glucolacto-oligosaccharides.
